# Explaining People’s Worry Levels During the Covid-19 Pandemic: An Analysis of Socio-Economic and Cultural Dimensions

**DOI:** 10.3389/fpsyg.2021.737917

**Published:** 2021-10-06

**Authors:** José I. Rojas-Méndez

**Affiliations:** Sprott School of Business, Carleton University, Ottawa, ON, Canada

**Keywords:** Covid-19 pandemic, worries, cultural dimensions, long-term orientation, uncertainty avoidance, income

## Abstract

This paper examines the influence of socio-economic and cultural dimensions (measured at the country level) on what concerns people the most about the Covid-19 pandemic. Based on secondary data, the study considers the opinion of more than 24,000 individuals living in 30 different countries, with national samples weighted to match each country’s general population older than 18years of age. A set of linear Bayesian regressions was applied to 10 different types of worries reported for economic, health, and safety domains. Results demonstrate that socio-economic variables and cultural dimensions complement each other in explaining people’s concerns about the effect of the Covid-19 pandemic. An overall view of the analysis also reveals that cultural dimensions exceed socio-economic variables in explaining peoples’ worries about health and safety domains. Socio-economic variables are slightly more effective in explaining the worries of the economic domain. Among the cultural dimensions, long-term orientation and uncertainty avoidance are the best in explaining people’s worries. The higher the score in long-term orientation, the lower the worry levels expressed by the respondents. Likewise, low scores on uncertainty avoidance generate lower levels of worries due to the Covid-19 pandemic. Finally, health worries produce a positive outcome because they explain a significant reduction in the fatality rate.

## Introduction

Worry is a normal phenomenon that affects us all, but probably what we are living through today is by far the most important worry our society has seen during our life span. The world has been facing the largest public health crisis in recent history (Schaw, 2020). Since December 2019, Covid-19 has infected and killed millions of people around the world. This new reality is causing individuals to be worried about their own circumstances and that of many others around them.

A worry has been defined as a disturbing cognition that a state of an object (i.e., my personal health, my country’s economic situation, etc.) in some domain of life (i.e., health, economic, social relations, etc.) will become discrepant from it desired state (Boehnke et al., 1998). This definition encompasses daily worries and prolonged, intense, and uncontrollable worries that might be associated with the clinical diagnosis of anxiety (Borkovec et al., 2004).

The current pandemic has worsened uncertainty not only over physical and mental health but also over the economy, employment, finances, and relationships. Every worry involves some potential danger to a personal or collective object in at least one of the six main domains of life identified by Boehnke et al. (1998): health, safety, environment, social relations, meaning in life, achievement in work and studies, and economics. Freeston et al. (1994) argue that individuals who worry do so because of a reduced ability to tolerate uncertainty. Specifically, in the context of worry, they introduced the concept of intolerance uncertainty by indicating that a primary reason individuals worry is to try to exert some control over situations they face in life with the hope of preventing or reducing future adverse outcomes. Researchers have demonstrated that worry and intolerance of uncertainty are positively related (Ladouceur et al., 2000).

Do we differ in how much worry and uncertainty we can tolerate in life? If so, what could explain the distinct worry levels experienced by people from different nations? Under normal circumstances, extant research demonstrates that people from diverse cultures react differently to different stimuli. Thus, this paper focuses on the influence of socio-economic and cultural dimensions on what concerns people most about the Covid-19 pandemic. Are cultural dimensions able to somehow explain how groups of individuals report their worries about the implications of Covid-19? Is the way individuals react to Covid-19 better explained by socio-economic conditions in the country they are living? Are worries producing a negative outcome under a pandemic situation like Covid-19? These are the main questions addressed in this paper, which to the best of our knowledge, is the first in addressing them. Specifically, we examine the interaction effects of socio-economic and cultural variables on 10 personal and collective concerns that people from 30 different countries may have related to the current Covid-19 pandemic. This study’s importance relies on providing some explanations to anticipate the degree of people’s concerns and consequences of a disturbing event like a pandemic. MacLeod et al. (1991) argue that worry is a cognitive phenomenon affected by future events coming with uncertainty about potential outcomes, creating anxiety feelings. Research has demonstrated that worry serves to exacerbate anxiety (Dickson et al., 2012). Thus, most of the time worries are associated with adverse outcomes, at least for mental health. However, the impact of worries may be different under a crisis like a pandemic.

The countries covered in this research are quite diverse in terms of socio-economic and cultural conditions. Geographically, they come from The Americas, Europe, Australasia, Asia, Africa, and the Middle East. They were measured almost simultaneously during late March and April 2020.

## Theoretical Framework

This study borrows from Rumination’s theory (Martin and Tesser, 1989) to explore if and why individuals show distinct levels of worry when facing similar events like a pandemic. Rumination theory postulates that individuals more disposed to ruminate tend to overemphasize negative information related to what they are experiencing and thus seeing problems as more threatening than they are (Lyubomirsky et al., 1999). Rumination and worry are significantly correlated with each other (Fresco et al., 2002; Watkins et al., 2005), and they share many characteristics (McLaughlin et al., 2006). For instance, both are repetitive, perseverative forms of thought that are self-focused (Borkovec et al., 2004) and are associated with difficulty in switching attention from negative stimuli (Hazlett-Stevens, 2001).

Nonetheless, rumination and worry differentiate from each other in the time orientation dimension. Worry tends to be future-oriented and usually focuses on threats that might occur but have not yet happened. In contrast, although rumination can also involve concerns about possible threats in the future, it primarily entails reminiscing over past events with a negative undertone (Nolen-Hoeksema et al., 2008).

Therefore, this research tests the interaction of socio-economic and cultural dimensions as potential explanatory variables for differences in the level of peoples’ worries or concerns reported during the global crisis caused by the Covid-19 pandemic. Besides, it also analyzes the impact of such worries on the number of deaths and fatality rates due to Covid-19 pandemic.

### Socio-Economic Influences

Having a high income allows people to stop worrying or worry less about survival and material matters. This is what Li et al. (2016) call the “money buffer effect.” In contrast, previous research has hypothesized that economic insecurity would increase people’s economic worries (Roth et al., 2017). Income, education, and material possessions, among other variables, are often considered essential resources to allow individuals to advance in society by achieving diverse goals in life. Diener et al. (1995) suggest that economic variables may contribute to worry by blocking or enhancing people’s ability to achieve their goals. Thus, poor economic country conditions may deprive its citizens of the means needed to satisfy their goals in life. In addition, national economic problems tend to increase the existence of difficulties among the majority in national society, probably extensively covered by the media, thus capturing people’s attention and producing higher worrying levels. Consequently, these societies are expected to have a higher level of a worry than more affluent nations. On the contrary, when economic resources are available, people can use them to reduce threats and worries to their well-being to a more desirable level. Thus, the following hypothesis is put forward:

*H1: Country's per capita income negatively affects the worry levels experienced by individuals regarding the Covid-19 pandemic.*

Employment rates currently available in each country may also influence the way individuals perceive distinct types of worry. In his classic study about cultural dimensions, Hofstede (2001) reports strong negative correlations between individuals’ stress levels at work and perceived employment stability in the future. More recently, Marlar (2010) says that unemployment is positively related to experience worry, and that this worry intensifies with the length of unemployment condition. Therefore, we propose the following hypothesis:

*H2: Country's employment rate negatively influences the worry levels experienced by individuals regarding the Covid-19 pandemic.*

### Cultural Dimensions Influence

Culture is defined as “a collective programing of the mind which distinguishes one group from another” (Hofstede, 1980, p. 25). The model of cultural dimensions proposed by Hofstede (2001) considers country citizens as a group of individuals who share a set of core values and practices theorized to transfer, influence, and normalize what people believe and how they think and behave (Noort et al., 2016). The extant literature is very generous in showing the link between cultural dimensions and people’s behavior and perceptions (e.g., Bolton et al., 2010; Sharma, 2010; Thompson and Chmura, 2015), but very few researchers have focused on the impact of culture on people’s concerns and worries, particularly under stressing situations like a pandemic. In a study about worries people commonly experience in their daily life (i.e., not under specific events like a pandemic), Schwartz and Melech (2000) demonstrate that worries have consistent meanings across cultures. Therefore, it is possible to make some broader inferences about the impact of cultural dimensions on different types of worries. This study uses five of Hofstede’s national cultural dimensions: power distance (PDI), individualism/collectivism (IND), masculinity/femininity (MAS), uncertainty avoidance (UAI), and long-term orientation (LTO). These five cultural dimensions have been selected as they are the most widely accepted drivers of the behavior of which there are scores for the most part of countries.

PDI is the “extent to which the less powerful members of a society expect and accept that power is distributed unequally” (Hofstede, 2001, p. 98) PDI is an indicator of social hierarchy, respect, wealth, rights and privileges (Sharma, 2010; Song et al., 2018). Low PDI cultures see individuals as moral equals who share primary interests as human beings, where their personal responsibility should transcend their selfish interests (Schwartz and Melech, 2000). This understanding implies the existence among low PDI societies of a natural tendency to voluntarily cooperate and feel concerned for other individuals’ welfare in their community. Consequently, these societies put aside selfish interests and worry more about societal problems than personal ones. High PDI nations emphasize a hierarchical system with tight control and unequal distribution of power, roles, and resources to ensure socially responsible behavior (Schwartz and Melech, 2000). As a result of this, individuals in low PDI societies are expected to worry more about themselves and those under their care, rather than the greater society. Consequently, the following hypothesis is proposed:

*H3: PDI positively impacts the worry levels of individuals regarding the Covid-19 pandemic.*

The cultural dimension of IND indicates that societies value independence and define the self according to individuals’ distinctive characteristics, and therefore ties between individuals are loose (Markus and Kitayama, 1991; Hofstede, 2001). In contrast, collectivistic cultures tend to define the self in terms of relationships with others, especially within-groups, emphasizing interdependence, in-group cohesiveness, attention to other peoples’ needs, and giving value to social harmony (Song et al., 2018). Collectivism closely links individuals to be part of groups such as family, peers, and society (Triandis, 1995; Cho et al., 2013). De Mooij and Hofstede (2011) posit that it is necessary for individuals to build a relationship that is conducive to trust in collectivistic cultures. As a result, after some time, individuals develop interest and even feelings and worries for an extended group of other people. Collectivistic nations stress maintaining the status quo and circumventing actions that might disrupt the solidarity among group members. Collectivists tend to prioritize group goals over personal interests and benefits; thus, they are more concerned for the public good (Sharma, 2010; Nguyen et al., 2017). On the contrary, individualistic societies allow individuals to pursue their own goals and not worry too much about others in the broader community, even when these are suffering (Schwartz and Melech, 2000). IND may provide a plausible explanation for the pressure Americans are putting on their government to re-open the economy as soon as possible. People in IND societies logically are expected to worry about their own health, safety and income and may not be naturally inclined to pay attention to other individuals’ problems because it is not necessarily normative to do that in individualistic cultures. Thus, the following hypothesis is suggested:

*H4: IND negatively impacts the worry levels of individuals regarding the Covid-19 pandemic.*

MAS has been described as a permanent pursuit of achievement and success, while the dominant values of a feminine culture are caring for other people and pursuing harmony and quality of life (De Mooij and Hofstede, 2011). MAS societies somehow emphasize personal things and divert the attention from problems in the broader society by showing a less-caring attitude for the weak (Song et al., 2018). In contrast, feminine cultures are more communal, emphasizing harmony among individuals, emphasizing getting along well with others, and paying attention to events that might threaten the wider society, which translates into lessening the importance of more personal worries. Hence, the following hypothesis is formulated:

*H5: MAS is expected to negatively influence the worry levels of individuals regarding the Covid-19 pandemic.*

UAI has been explained by the extent to which individuals feel threatened by situations that are accompanied by uncertainty and ambiguity (Hofstede, 2001). Hofstede et al. (2010) suggest that UAI indicates peoples’ anxiety level toward an ambiguous, unpredictable, and uncertain future. Other researchers conceptualize this cultural dimension as individuals’ dependence on implicit or explicit rules, structures, and relationships to get away from the ambiguity experienced in everyday life (Yaveroglu and Donthu, 2002). Countries exhibiting high UAI are less tolerant of unorthodox behaviors and ideas (Song et al., 2018). In countries of strong UAI, people prefer to maintain clarity and adhere to the status quo (Sharma, 2010). If the status quo is challenged, for instance, by a pandemic, they require rules and formality to structure life under the new uncertain conditions. Countries like Italy (UAI=75) and Spain (UAI=86), which score high in this dimension, have demonstrated this by imposing a state of emergency, quarantine, etc., as a mean to control the spread of Covid-19. In contrast, countries weak in UAI, such as Sweden (UAI=29), have released only recommendations to its citizens to keep the social distance. Similarly, individuals living in the latter type of countries like United States (UAI =46) feel uncomfortable with newly imposed restrictions and demand them to be lifted as soon as possible. Consequently, the following hypothesis is postulated:

*H6: UAI exercises a positive effect upon the worry level of individuals regarding the Covid-19 pandemic.*

Finally, LTO is also expected to exercise some influence on people’s concerns about Covid-19 pandemic. Individuals living in societies scoring high in this dimension, perhaps influenced by Confucian ethics, are more perseverant, socially conscious and patient than their counterparts from short term-oriented societies (Steenkamp et al., 1999; Sharma, 2010). Perseverance has been explained “as the conscientiousness that is required to persist over time. It is based on the belief that efforts made today will pay off in the future. Perseverance is needed for mere survival, but its cumulative effect is value-creating; it takes time for some things to gain value.” (Lumpkin and Brigham, 2011, p. 1154). LTO societies discount the future and value immediate results much less than short-term oriented cultures (Chen et al., 2005). LTO societies are generally persistent, dynamic in thinking and willing to accept radical changes, and therefore more likely to overcome uncertainties (Sharma, 2010; Nguyen et al., 2017). In contrast, short-term oriented ones are expected to solve issues faster, increasing personal steadiness and stability and enjoying the “here and now” (De Mooij and Hofstede, 2011; Song et al., 2018). Short-term orientation relates with stability and focus on the past or the present (Donthu and Yoo, 1998), whereas LTO implies viewing time holistically, valuing both the past and the future, rather than deeming actions necessary only for their effects in the here and now (Bearden et al., 2006). As a result, the following hypothesis is offered:

*H7: LTO has a negative impact on the worry levels of individuals regarding the Covid-19 pandemic.*

## Methodology

### Data and Measures

Independent socio-economic variables used in this study are *per capita* income estimated in US$ purchasing power parity for 2020 (IMF, 2020) and 2019 employment rate (Euromonitor, 2020) for each country considered. The other independent variables (i.e., cultural dimensions) were taken from Hofstede (2020), which are represented in normalized scores ranging from 0=low level and 100=high level of presence of the corresponding dimension. The data on worries or concerns (i.e., dependent variables) were taken from the Consumer Pulse Survey of McKinsey (2020). They collected data at the end of March and April 2020 in 30 different countries, with samples considered representative since they weighted them to match each country’s general population older than 18years of age. The general question presented to respondents was: What concerns you most about the Covid-19 situation? Respondents were asked to declare their level of concern about 10 types of worries on a 5-point Likert scale where 1=not a concern, and 5=extremely concerned. The actual data used for the current study in each of the 10 dependent variables relate to the % of respondents who are very concerned or extremely concerned (i.e., points 4 and 5 in the Likert scale). Table 1 shows the list of countries, their corresponding sample sizes, and the aggregated values for the dependent and independent variables used in this study.

**Table 1:** Countries considered in the study (in alphabetical order) and values for the dependent and independent variables.

Country	Sample size	Independent variables							Dependent variables: worries			
		Socio-economic		National cultural dimensions					Economic	Health	Safety	Overall
		Income	Employment Rate	Power distance (PDI)	Individualism/collectivism (IND)	Masculinity/femininity (MAS)	Uncertainty avoidance (UAI)	Long-term orientation (LTO)				
Argentina	1,007	19970.52	61.7	49	46	56	86	20	61.50	60.00	74.50	63.50
Australia	669	54799.04	74.1	38	90	61	51	21	41.00	47.50	49.50	45.30
Belgium	604	50904.69	65.2	65	75	54	94	82	30.00	46.75	48.50	40.40
Brazil	1,013	17016.32	64.4	69	38	49	76	44	64.00	72.00	75.00	69.40
Canada	1,034	52144.45	73.5	39	80	52	48	36	45.25	55.00	54.50	51.00
Chile	1,005	27150.38	60.0	63	23	28	86	31	70.75	73.25	87.50	75.10
China	1,216	20984.28	75.5	80	20	66	30	87	21.25	21.75	25.50	22.30
Colombia	1,005	16264.97	61.1	67	13	64	80	13	68.00	70.00	79.50	71.10
Denmark	603	55675.00	75.1	18	74	16	23	35		34.25	32.00	
France	1,003	48640.05	66.6	68	71	43	86	63	34.50	49.50	49.50	43.50
Germany	1,002	55306.21	76.0	35	67	66	65	83	28.75	36.50	30.50	32.20
India	601	9026.87	43.6	77	48	56	40	51	67.50	76.00	83.50	74.10
Indonesia	722	14840.76	65.5	78	14	46	48	62	46.25	47.75	54.00	48.40
Italy	1,009	41582.19	58.7	50	76	70	75	61	50.25	56.50	58.00	54.30
Japan	600	46827.35	77.9	54	46	95	92	88	44.75	41.50	38.50	42.20
Mexico	1,506	21363.00	61.7	81	30	69	82	24	76.75	60.75	82.50	71.50
Nigeria	531	6171.68	53.3	80	30	60	55	13	73.25	81.50	86.00	79.10
Peru	1,012	15398.62	68.7	64	16	42	87	25	58.00	71.25	81.50	68.00
Poland	607	35651.18	62.2	68	60	64	93	38	56.25	74.00	75.50	67.20
Portugal	601	34935.82	69.4	63	27	31	99	28	62.50	78.00	81.50	72.50
Saudi Arabia	510	56912.37	54.9	95	25	60	80	36	34.25	48.25	52.00	43.40
South Africa	535	13965.17	42.0	49	65	63	49	34	76.00	77.00	82.00	77.60
South Korea	600	46451.61	65.2	60	18	39	85	100	35.75	30.25	44.50	35.30
Spain	1,006	43007.50	63.4	57	51	42	86	48	60.50	78.75	85.50	72.80
Sweden	201	55988.99	77.5	31	71	5	29	53	30.00	41.00	36.50	35.70
Switzerland	745	67557.70	79.0	34	68	70	58	74	27.00	34.50	34.00	31.40
Turkey	599	29327.00	48.4	66	37	45	85	46	45.00	58.00	42.00	49.60
United Arab Emirates	510	70441.55	81.2	90	25	50	80		56.50	66.75	71.50	63.60
United Kingdom	1,005	48168.87	74.4	35	89	66	35	51	42.00	55.75		
United States	1,063	67426.84	68.8	40	91	62	46	26	47.75	56.00	58.50	53.20
Total Sample	24,124	19970.52	61.7	49	46	56	86	20	50.18	56.67	60.48	55.49

### Method

In search for a more aggregated way to present the results of this study, the 10 worries included as dependent variables were subjected to Exploratory Factor Analysis with a non-orthogonal rotation (i.e., Direct Oblimin). Results confirm the presence of only one factor extracting 86% of the total variance. Cronbach alpha for this index achieves 0.98, which is deemed excellent. Consequently, an overall worry index was created as the average of the 10 different types of worries. Nevertheless, based on face validity, the worries were subclassified into three types to better represent their nature: economic, health, and safety. These also achieved excellent levels as indicated by their Cronbach’s alpha values: Economic Worries (4-item, 0.96), Health Worries (4-item, 0.95), and Safety Worries (2-item, 0.96). Thus, these aggregated dependent variables were added as the average of their composing variables.

Table 2 shows the correlation coefficients among independent and dependent variables used in this study. The results of this primary step allow anticipating some impact of the independent over the dependent variables. For instance, the socio-economic variables are significantly correlated with all types of worries. In turn, cultural values show some significant correlations, particularly PDI, UAI and LTO.

**Table 2:** Correlation matrix.

Variables	Socio-economic		National cultural dimensions					Worries			
	Income	Employment rate	PDI	IND	MAS	UAI	LTO	Economic	Health	Safety	Overall
Income											
Employment Rate	0.636^**^										
PDI	−0.435^*^	−0.401^*^									
IND	0.548^**^	0.224	−0.731^**^								
MAS	−0.053	−0.060	0.165	0.089							
UAI	−0.072	−0.163	0.425^*^	−0.385^*^	0.139						
LTO	0.328	0.332	−0.057	0.057	0.160	−0.002					
Economic worries	−0.629^**^	−0.559^**^	0.293	−0.323	−0.010	0.240	−0.697^**^				
Health worries	−0.501^**^	−0.555^**^	0.332	−0.207	−0.041	0.342	−0.675^**^	0.884^**^			
Safety worries	−0.563^**^	−0.533^**^	0.411^*^	−0.342	−0.020	0.373^*^	−0.662^**^	0.935^**^	0.939^**^		
Overall worries	−0.567^**^	−0.554^**^	0.294	−0.262	−0.096	0.271	−0.735^**^	0.969^**^	0.969^**^	0.978^**^	

* *Significant at p≤0.05.*

** *Significant at p≤0.01.*

Next, our analysis focused on testing whether socio-economic and cultural variables may be used to predict worry levels. To accomplish that, linear Bayesian regressions in SPSS 26 with non-informative prior distribution were used. The selection of non-informative prior is used due to the great deal of uncertainty in the population parameter. The rationale for choosing Bayesian regression over traditional frequentist methods has been identified by van Schoot and Depaoli (2014). This method is appropriate for parameter estimation with limited information available (Rossi and Allenby, 2003; Carlin and Louis, 2010; Sapsis, 2020) in (1) complex models, (2) when the researcher prefers the definition of probability, (3) when background knowledge can be incorporated into the analysis, and (4) only a small sample is available. Despite the number of individuals sampled in the current study (i.e., 24,000+), all data is aggregated at a country level for each of the 30 countries included, so our analysis is based on 30 observed cases. Thus, the Bayesian point estimator is the mean of the posterior distribution obtained after analysis. This resulting estimator is dominated by sample information (Greene, 2012).

The Bayesian model allows placing the parameters in a credible interval (95%), assuming that these parameters are random, unlike the frequency methods that give a point estimator. Wagenmakers et al. (2018, p. 38) explain the difference between confidence interval and credible interval as follow: “An X% confidence interval for a parameter θ is an interval generated by a procedure that in repeated sampling has an X% probability of containing the true value of θ. Thus, the confidence in the classical confidence interval resides in its performance in repeated use, across hypothetical replications. In contrast, the confidence in the Bayesian credible interval refers directly to the situation at hand.” For example, A 95% credible interval ranges from 0.18 to 0.59, which means that one can be 95% confident that the true value of ρ lies between 0.18 and 0.59. In this research, the data come from different countries; therefore, there could be effects of varying magnitude of the explanatory variables on the model’s dependent variables, depending on the country. To capture this heterogeneity in small sample sizes, as in this study, it makes more sense to place the random parameters in a credible interval (Bayesian approach) rather than determining a point estimator that may be biased (parametric multiple linear regression).

The estimator obtained by Bayesian methods can be interpreted in the same way as the estimators by classical approaches as if they were maximum likelihood estimators (Train, 2009). To calculate the Bayesian estimator (i.e., the mean of the posterior distribution), it is necessary to use simulation techniques (Train, 2009). In this case, random values were extracted from the posterior distribution (Gelman and Rubin, 1992) to allow inference from Bayesian estimators.

Using the available observations, two socio-economic variables (i.e., *per capita* income and employment rate) and five cultural dimensions (i.e., power distance, individualism/collectivism, masculinity/femininity, uncertainty avoidance, and long-term orientation) have been specified.

A maximum number of 10,000 iterations were established for each model, and measures of model fit based on the Bayesian factor, the value of adjusted *R*^2^, and the model’s overall significance were obtained. The Bayes’ factor contrasts the hypothesis of strength in evidence provided by the sample information to support the proposed model (Kass and Raftery, 1995). It contrasts the hypothesis that the model resulting from Bayesian estimation is preferable to the model that tests the null hypothesis. The scale proposed by Jeffreys (1961) will be used to assess the magnitude of the Bayesian factor. The credible intervals were estimated at 95% confidence; therefore, for the significance comparison of each parameter, it was considered that if 0 is within the credible interval, then the null hypothesis that the estimator is nonzero cannot be rejected, and therefore, it will not be significant at 5%.

## Results

Results are presented based on the Bayesian regressions for each of the dependent variables used to measure worries or concerns in samples from the 30 countries. In all models run, the measures of goodness of fit are satisfactory, the measure of *R*^2^ (as displayed by SPSS software) is at an adequate level, and the factor of Bayes >100 indicates that the robustness of the information in the sample supports the proposed model decisively (models are significant at 1%). The estimators and measures of fit for each Bayesian regression model are detailed in Table 3. The variance of the estimator and the credible intervals at 95% are reported in Table 4.

**Table 3:** Bayesian regression analysis, worries indicators as dependent variables.

	Independent variables							Adjusted *R*^2^			Model fit	
	Demographics		Cultural dimensions									
Dependent variables	Income US$ PPP	Employment Rate	Power distance	Individualism	Masculinity	Uncertainty avoidance	Long-term orientation	Total	Socio-economic	Cultural dimensions	Bayes factor	Sig.
**Economic Worries**	**0.000**	**−0.402**	**−0.273**	**−0.006**	**0.085**	**0.184** ^*^	**−0.298** ^*^	**0.752**	**0.397**	**0.355**	**6109.758**	**< 0.001**
1. The country’s economy	−0.610^*^	−0.489	−0.426^*^	0.225	0.067	0.383^*^	−0.185^*^	0.656	0.247	0.409	350.333	< 0.001
2. Not being able to get the supplies I need	−0.613^*^	−0.045	−0.070	0.040	0.139	0.121	−0.367^*^	0.719	0.435	0.284	1777.115	< 0.001
3. Negative impact on my job or income	−0.422^*^	−0.459	−0.180	−0.123	0.163	0.190^*^	−0.333^*^	0.788	0.410	0.378	54971.990	< 0.001
4. Not being able to make ends meet	−0.392^*^	−0.519	−0.162	−0.040	0.123	0.176	−0.259^*^	0.709	0.430	0.279	2016.539	< 0.001
**Health Worries**	**0.000**	**−0.323**	**0.015**	**0.255**	**−0.084**	**0.283** ^*^	**−0.308** ^*^	**0.707**	**0.194**	**0.513**	**1901.387**	**< 0.001**
5. Overall public health	−0.661^*^	−0.065	0.007	0.375^*^	−0.025	0.292^*^	−0.350^*^	0.608	0.197	0.411	89.634	< 0.001
6. Health of my relatives in vulnerable populations	−0.353	−0.255	−0.048	0.286	−0.175	0.333^*^	−0.341^*^	0.628	0.100	0.528	155.030	< 0.001
7. My personal health	−0.371	−0.731^*^	−0.009	0.158	0.064	0.174	−0.280^*^	0.630	0.229	0.401	163.469	< 0.001
8. Contributing to spread the virus	−0.467^*^	−0.258	0.110	0.203	−0.200	0.334^*^	−0.260^*^	0.703	0.158	0.545	1625.515	< 0.001
**Safety Worries**	**−0.001**	**−0.277**	**0.035**	**0.147**	**−0.078**	**0.332** ^*^	**−0.379** ^*^	**0.698**	**0.143**	**0.555**	**859.625**	**< 0.001**
9. Safety of myself or my family	−0.428^*^	−0.440	−0.041	0.130	−0.022	0.322^*^	−0.335^*^	0.726	0.177	0.549	2228.434	< 0.001
10. Taking care of my family	−0.643^*^	−0.055	0.112	0.201	−0.111	0.320^*^	−0.424^*^	0.630	0.119	0.511	166.953	< 0.001

* *Coefficient is significant at the 0.05 level*.

**Table 4:** Ninety five percent credible interval and Bayesian estimator variance.

	(1)			(2)			(3)			(4)			(5)		
	95% cred. Inter.			95% cred. Inter.			95% cred. Inter.			95% cred. Inter.			95% cred. Inter.		
	Lower	Upper	Var	Lower	Upper	Var	Lower	Upper	Var	Lower	Upper	Var	Lower	Upper	Var
Income PPP US$ 2020	−0.986	−0.234	0.036	−0.984	−0.243	0.035	−0.744	−0.100	0.026	−0.731	−0.054	0.029	−1.103	−0.220	0.050
Employment rate 2019	−1.074	0.096	0.088	−0.619	0.528	0.084	−0.960	0.042	0.064	−1.045	0.008	0.071	−0.752	0.622	0.121
Power distance	−0.811	−0.040	0.038	−0.461	0.321	0.039	−0.511	0.150	0.028	−0.509	0.185	0.031	−0.446	0.460	0.052
Individualism	−0.072	0.523	0.023	−0.255	0.336	0.022	−0.378	0.132	0.017	−0.308	0.228	0.018	0.025	0.724	0.031
Masculinity	−0.178	0.312	0.015	−0.109	0.386	0.016	−0.047	0.373	0.011	−0.097	0.344	0.012	−0.312	0.263	0.021
Uncertainty avoidance	0.181	0.585	0.010	−0.084	0.326	0.011	0.017	0.363	0.008	−0.006	0.358	0.008	0.055	0.529	0.014
Long Term orientation	−0.369	−0.002	0.009	−0.547	−0.187	0.008	−0.490	−0.176	0.006	−0.424	−0.094	0.007	−0.565	−0.135	0.012
	(6)			(7)			(8)			(9)			(10)		
Income PPP US$ 2020	−0.733	0.026	0.037	−0.794	0.053	0.046	−0.824	−0.110	0.033	−0.796	−0.059	0.035	−1.155	−0.132	0.067
Employment Rate 2019	−0.846	0.355	0.089	−1.373	−0.054	0.111	−0.813	0.298	0.079	−1.016	0.135	0.085	−0.851	0.742	0.162
Power Distance	−0.437	0.341	0.039	−0.443	0.426	0.048	−0.256	0.476	0.034	−0.417	0.334	0.036	−0.413	0.637	0.070
Individualism	−0.015	0.586	0.023	−0.178	0.493	0.029	−0.079	0.486	0.020	−0.164	0.423	0.022	−0.205	0.606	0.042
Masculinity	−0.422	0.072	0.016	−0.212	0.340	0.019	−0.433	0.032	0.014	−0.263	0.218	0.015	−0.444	0.223	0.028
Uncertainty Avoidance	0.129	0.536	0.011	−0.054	0.401	0.013	0.142	0.525	0.009	0.123	0.520	0.010	0.045	0.594	0.019
Long Term Orientation	−0.526	−0.156	0.009	−0.487	−0.074	0.011	−0.434	−0.086	0.008	−0.514	−0.157	0.008	−0.673	−0.174	0.016

*The first column lists all the independent variables. The 10 additional columns correspond to each of the dependent variables signaled by numbers from 1 to 10 in the bottom part of* *Table 3*.

To facilitate a more aggregated view of the results, the 10 different worries considered in this study have been classified into three main domains of life identified by Boehnke et al. (1998): economic (4), health (4), and safety (2).

### Economic Worries

#### Worry for the Country’s Economy

The average *per capita* income (*β*=− 0.610; *p*≤0.05) is a significant negative factor explaining this worry, which means that an increase in income reduces concern for the national economy situation. At the same time, three cultural dimensions have a significant impact on this worry. UAI has a positive effect (*β*=0.383; *p*≤0.05); whereas PDI (*β*=− 0.426; *p*≤0.05) and LTO (*β*=− 0.185; *p*≤0.05) resulted in significative negative influence. An increase in PDI and LTO produces a decrease in concern for the country’s economy; conversely, a greater aversion to uncertainty produces a greater concern for the national economy. Altogether, the independent variables explain a considerable level of the variance of worry for the country’s economy (Bayesian *R*^2^=0.66).

#### Not Being Able to Get the Supplies I Need

Only two variables were significant in this type of worry, and both were producing a negative impact on the dependent variable: Income (*β*=−0.613; *p*≤0.05) and LTO (*β*=− 0.367; *p*≤0.05). This negative coefficient for income indicates that people will reduce their concern for this worry as income increases in a country. Similarly, as LTO increases, the level of worry moves in the opposite direction. The general model explains a sizable portion of the dependent variable variance (Bayesian *R*^2^=0.72).

#### Negative Impact on My Job or Income

Income inversely affects the concern for a negative impact on the work or income (*β*=−0.422; *p*≤0.05), suggesting that an increase in income reduces this concern. With respect to culture, two dimensions were significant: UAI (*β*=0.190; *p*≤0.05) and LTO (*β*=− 0.333; *p*≤0.05). These coefficients indicate, on the one side, that the higher a country scores in UAI, the more people will worry about the negative impact of the Covid-19 pandemic on their job and income. On the other side, short-term oriented people tend to express more worries about the negative effects of the Covid-19 pandemic on the job and income. Overall, the independent variables’ explanatory power on the negative impact on one’s job or income is the highest among all regression models run (Bayesian *R*^2^=0.79).

#### Not Being Able to Make Ends Meet

Income is impacting in a significant and negative way the concern of not being able to pay the monthly expenses (*β*=−0.392; *p*≤0.05). An increase in income reduces this concern. Concerning cultural dimensions, only LTO is significant (*β*=− 0.259; *p*≤0.05), meaning that short-term oriented societies tend to worry more about not having enough to cover monthly expenses. The total variance explained by income and long-term orientation in the dependent variable is also very good (Bayesian *R*^2^=0.71).

Overall, the economic worries achieve an adjusted Bayesian *R*^2^=0.75 when using socio-economic and cultural variables as independent variables. To estimate each set of independent variables’ partial contribution, a Bayesian multiple regression with a stepwise approach was used. Results indicate that 0.40 is contributed by the socio-economic variables and 0.35 from cultural dimensions.

### Health Worries

#### Overall Public Health

In general, public health concern is significantly impacted by income (*β*=−0.661; *p*≤0.05), signifying that an increase in its value reduces public health concern. In terms of culture, three dimensions are significant: IND (*β*=0.375; *p*≤0.05), UAI (*β*=0.292; *p*≤0.05) and LTO (*β*=−350; *p*≤0.05). Greater IND, higher UAI and short-term orientation lead to greater concern for public health. All in all, this regression model explains a substantial part of the dependent variable (Bayesian *R*^2^=0.61).

#### Health of My Relatives in Vulnerable Populations

Interestingly, only the cultural dimensions of UAI (*β*=0.333; *p*≤0.05) and LTO (*β*=−0.341; *p*≤0.05) are significant in the model. A higher level of UAI increases the concern for relatives in the population at risk, and a greater LTO produces a decrease in this concern. Even though only two variables were significant, an important level of the total variance in the dependent variable was achieved (Bayesian *R*^2^=0.63).

#### My Personal Health

This worry is the only one where the country’s employment rate achieves a significant role in explaining the dependent variable (*β*=−0.731; *p*≤0.05). The national employment rate has a significant influence on reducing personal health concerns. Regarding culture, LTO is the only one achieving a significant level in the standardized coefficient (*β*=−0.280; *p*≤0.05), pointing out that a greater LTO reduces personal health worries. As has been the case in all regressions explained above, this model reaches a good explanatory level (Bayesian *R*^2^=0.63).

#### Contributing to Spread the Virus

This dependent variable decreases significantly as the country’s income increases (*β*=−0.467; *p*≤0.05). The cultural dimensions that significantly influence this worry are UAI (*β*=0.334; *p*≤0.05) and LTO (*β*=− 0.260; *p*≤0.05). The regression model shows that a higher level of UAI and a short-term orientation increase the concern for spreading the virus. Overall, the explanatory level in the dependent variable is also very good (Bayesian *R*^2^=0.70).

In summary, the health worries achieve an adjusted Bayesian *R*^2^=0.71 when using socio-economic and cultural variables as independent variables. To estimate the partial contribution of each set of independent variables, a Bayesian multiple regression with a stepwise approach was run. Results indicate that 0.20 is contributed by the socio-economic variables and 0.51 from cultural dimensions.

### Safety Worries

#### Safety of Myself or My Family

Income is a negative and significant variable explaining concern for oneself or one’s family (*β*=− 0.428; *p*≤0.05), thus suggesting that an increase in the average income reduces this concern. With respect to cultural dimensions, two variables were significant: UAI (*β*=0.322; *p*≤0.05) and LTO (*β*=−0.335; *p*≤0.05). Scoring higher in UAI and being short-term oriented contributes to a society worrying more about oneself and family’s safety. The general model explains a significant level of variance of the dependent variable (Bayesian *R*^2^=0.73).

#### Taking Care of My Family

In this model, income achieves a significant negative coefficient (*β*=−0.643; *p*≤0.05). An increase in income reduces the concern for the family. With respect to culture, the same two dimensions were significant: UAI (*β*=0.320; *p*≤0.05) and LTO (*β*=−0.424; *p*≤0.05). Again, scoring high in UAI and being short-term oriented make society worry more about taking care of one’s family. Overall, the model also explains a very good level of the variance in the dependent variable (Bayesian *R*^2^=0.63).

Altogether, the safety worries achieve an adjusted Bayesian *R*^2^=0.70 when using socio-economic and cultural variables as independent variables. To estimate each set of independent variables’ partial contribution, a Bayesian multiple regression with a stepwise approach was used. Results indicate that 0.14 is contributed by the socio-economic variables and 0.56 from cultural dimensions.

### Are Worry Levels Explaining Covid-19 Deaths?

To investigate the potential effect of the worries included in this study over what many may consider the most significant outcome in this Covid-19 crisis (i.e., deaths), two new Bayesian regression analyses were modeled. The country-level indicators of the number of deaths per 100K population and the fatality rate over the total number of infected people per country as of November 30, 2020, were used as dependent variables for these estimates (John Hopkins University, 2020). We argue that worries play the role of an antecedent of death per 100K and fatality rate because worry levels were measured 7months before those. Consequently, we are testing whether worry levels somehow impact the dependent variables after a few months.

Table 5 shows that the 95% credible intervals for the three independent variables do include 0, indicating that there is no effect in predicting death per 100K. The regression model for deaths per 100K inhabitants explains only 12% of its variance but is not significant, probably due to the omitted variable bias.

**Table 5:** Bayesian Estimates

A: Bayesian estimates of coefficients for death per 100K as dependent variable					
Parameter	Posterior			95% credible interval	
	Mode	Mean	Variance	Lower bound	Upper bound
Intercept	21.549	21.549	795.267	−34.176	77.274
Health_Worries_Index	−0.372	−0.372	1.733	−2.974	2.230
Eco_Worries_Index	−2.459	−2.459	1.794	−5.106	0.187
Safety_Worries_Index	2.819	2.819	2.078	−0.030	5.667
Error Variance^#^	1357.569	1604.399	257409.757	896.675	2846.251
**B: Bayesian estimates of coefficients for case fatality as dependent variable**					
Intercept	3.211	3.211	1.109	1.130	5.293
Health_Worries_Index	−0.142^*^	−0.142	0.002	−0.239	−0.045
Eco_Worries_Index	0.082	0.082	0.003	−0.016	0.181
Safety_Worries_Index	0.056	0.056	0.003	−0.050	0.162
Error Variance^#^	1.894	2.238	0.501	1.251	3.971

# *Assume standard reference priors.*

* *Coefficient is significant at the 0.05 level*.

*A: Adjusted R^2^ =0.120, Bayes Factor=0.167, and Significance=0.112. B: Adjusted R^2^ =0.240, Bayes Factor=0.967, and Significance=0.022.*

For their part, as Table 5 indicates, the overall model for the fatality rate is significant, explaining 24% of the variance in the case fatality variable (Bayesian *R*^2^=0.24). However, there is no evidence that economic worries and safety worries are good predictors of the dependent variable. In turn, health worries are the only significant predictor, having a significant negative influence on the case fatality rate, which is a positive outcome for society. Results in Table 5 show that the posterior mean of the regression coefficients of health worries is −0.142, while for economic worries is 0.082 and for safety worries is 0.056. We can interpret this estimate such that one unit increase in the health worries leads to a decrease of 0.142units in the case fatality on average. The corresponding 95% credible interval of [−0.239, −0.045] indicates a 95% probability that the regression coefficient of health worries lies in the population within the corresponding credible interval. Also, the 95% credible interval does not include 0, so it is reasonable to conclude that health worries affect the prediction of case fatality.

So, being worried about health conditions seems to positively affect after a few months by reducing the number of casualties due to the pandemic. One possible explanation for this significant impact is that higher levels of health worries will probably mean that people will engage in more prosocial behavior. Serrano-Montilla et al. (2021) report that perceived threat is indirectly (*via* empathic concern) linked to prosocial tendencies. Among those prosocial tendencies, we may mention adopting more measures to avoid getting infected and disseminating the virus to others (reducing time spent outside the home, keeping social distance, wearing masks, and washing hands frequently). As a result of the prosocial behavior, it would be expected a lower level of fatality rate.

## Discussion and Implications

Table 6 summarizes the evidence from our work that is statistically significant against each hypothesis. Overall, there is enough evidence to fully support three hypotheses that deal with income, UAI and LTO upon the different types of worries. Two other hypotheses, one dealing with the socio-economic variable of the country’s employment rate and the other one focusing on PDI’s cultural dimension, received marginal support. Lastly, two hypotheses were not supported at all. MAS was non-significant in all the Bayesian regressions run in this study. Surprisingly, IND was significant in only one of the worries but in the opposite direction to the expected one (see more on this further down in the paper).

**Table 6:** Summary of hypotheses.

Hypotheses	Evidence
H1: Country’s *per capita* income negatively affects the worry levels experienced by individuals regarding the Covid-19 pandemic.	Supported in eight out of 10 Bayesian regressions. The two worries where H1 is not supported belong to the health dominion of worries: health of my relatives in vulnerable populations and my personal health.
H2: Country’s employment rate negatively influences the worry levels experienced by individuals regarding the Covid-19 pandemic.	Supported in only one out of 10 Bayesian regressions (My personal health).
H3: PDI positively impacts the worry levels of individuals regarding the Covid-19 pandemic.	Supported in only one out of 10 Bayesian regressions (The country’s economy).
H4: IND negatively impacts the worry levels of individuals regarding the Covid-19 pandemic.	Not supported. Surprisingly in one Bayesian regression, a significant coefficient is found in the opposite direction (Overall public health).
H5: MAS is expected to negatively influence the worry levels of individuals regarding the Covid-19 pandemic.	Not supported.
H6: UAI exercises a positive effect upon the worry levels of individuals regarding the Covid-19 pandemic.	Supported in seven out of 10 Bayesian regressions. The three worries where H6 is not supported are: Not being able to get the supplies I need, not being able to make the ends meet (both belonging to the economic dominion of worries), and my personal health.
H7: LTO has a negative impact on the worry levels of individuals regarding the Covid-19 pandemic.	Supported in 10 out of 10 Bayesian regressions.

This study contributes to our current knowledge of how people react in terms of worries caused by a crisis event like a pandemic. First, as proposed at the outset, Rumination theory is a good foundational support for the analysis of the potential impact on important outcomes such as deaths resulting from a pandemic. This theory posits that individuals more disposed to ruminate usually overemphasize negative information related to what they are experiencing and thus seeing problems as more threatening than they are (Lyubomirsky et al., 1999). However, a contribution from this study is that worry levels in the health domain result in a positive outcome for countries by reducing the fatality rate.

Second, socio-economic variables and cultural dimensions complement each other in explaining people’s concerns about the effect of the Covid-19 pandemic. Altogether, socio-economic variables and cultural dimensions explain between 61 and 79% of the variation on any of the worry levels considered in this study. The negative relationship between income and worries could be explained by the fact that, in higher-income economies, household savings levels are more likely to occur. Thus, people there may enjoy greater financial security. An overall view of the analysis makes evident that cultural dimensions as a group exceed socio-economic variables in explaining peoples’ worries due to the Covid-19 pandemic in the health and safety domain. As one may expect, socio-economic variables are slightly better than cultural variables in explaining worries classified in the economic realm. Higher scores in socio-economic variables may give people a powerful sense of safety and confidence, reducing the intensity of worries.

Third, the best explanatory cultural dimensions upon worries are LTO and UAI. LTO significantly reduces the degree of concern expressed by the respondents. Likewise, low UAI also generates lower levels of worries due to the Covid-19 pandemic. Somehow unexpected, the cultural dimensions of PDI, MAS, and IND are marginally or non-significant in explaining peoples’ concerns. One may speculate that PDI’s lack of impact upon worries is because none of its traditional assumptions can be fully developed under a pandemic crisis. On the one side, low PDI societies are expected to portray individuals as moral equals that transcend their selfish interests, so people may voluntarily cooperate with each other to improve the general welfare. On the other side, in high PDI cultures, people are required to meet role obligations that may contradict personal interests. However, under a pandemic situation, the freedom to decide what to do may be put aside, and governments’ decisions may be imposed to “all individuals,” no matter their role in society, to control the crisis. Besides, no matter the status in the community, all individuals feel vulnerable to potential adverse consequences in a pandemic situation. In the case of MAS, societies encourage mastering and changing the surrounding world to their will by overcoming any barrier that may appear. In contrast, feminine cultures try to preserve it and usually are less worried about what could be out of control. But under the current Covid-19 pandemic, for which there is not an effective treatment yet discovered, there seem to be no significant differences in the way societies worry about potential consequences, no matter if they can be classified as masculine or feminine. Lastly, a surprising finding is the absence of any negative effect of IND upon the worries reported by respondents. Above all was the discovery that IND impacts positively, not negatively as expected, on the worry of “overall public health.” As stated earlier, IND cultures value independence and define the self according to individuals’ distinctive characteristics, thus having loose ties with others, whereas collectivistic cultures explain the self in terms of relationships with others, emphasizing interdependence, in-group cohesiveness, attention to other peoples’ needs, and closely linking individuals through an interest in each other. It seems that a pandemic like Covid-19 permeates all theoretical considerations that are traditionally associated with IND and collectivism. Under a crisis like this, and until we have an effective antidote available, the individual may affect others, and the society may affect individuals with fatal consequences.

Overall, the main contribution in cross-cultural psychology can be the supporting evidence that the national cultural dimensions, specifically LTO and UAI, are suitable explanatory variables upon the worry levels included in this study. In turn, worry levels, particularly in the health domain, contribute positively to reducing the fatality rate under a pandemic like Covid-19. As such, worry levels are not always harmful as we usually see them. Consequently, governments may positively reduce their countries’ fatality rate by developing and implementing informative campaigns highlighting the potential health risks due to a critical situation like a pandemic.

## Limitations and Future Research

The first limitation of this study relates to the number of countries considered; although it is much larger than that of other studies dealing with worries (Schwartz and Melech, 2000), it represents around 15% of all the world countries. Including additional countries in future research may provide more evidence of the interaction between socio-economic and cultural variables and their impact on worries. Having a database with more observations (i.e., more countries) may allow for further analysis, such as testing to see whether worry levels mediate the relationship between socio-economic and cultural variables upon the death of people in a pandemic situation. For such a test, it is recommended sample sizes of at least 50 observations (Fritz and MacKinnon, 2007; Schoemann et al., 2017).

A second limitation relates to the independent variables used here. Other objective socio-economic measures may be included in future studies, such as the Gini coefficient, which reports income inequality. Likewise, cultural dimensions beyond those offered by Hofstede could be used, including additional ones proposed by The Globe Project, Schwartz and Trompenaars.

Third, the data used for all analyses in this study are at a country, not an individual level; thus, it is impossible to identify any variability within the sample. Also, Denmark, United Arab Emirates, and the United Kingdom presented missing values for one variable each.

Fourth, additional dependent variables, such as those related to mental health, may be used to test the effect of worry levels in the population.

## Data Availability Statement

The original contributions presented in the study are included in the article/supplementary material, further inquiries can be directed to the corresponding author.

## Ethics Statement

Ethical review and approval were not required for the study on human participants in accordance with the local legislation and institutional requirements. Written informed consent for participation was not required for this study in accordance with the national legislation and the institutional requirements.

## Author Contributions

The author confirms being the sole contributor of this work and has approved it for publication.

## Conflict of Interest

The author declares that the research was conducted in the absence of any commercial or financial relationships that could be construed as a potential conflict of interest.

## Publisher’s Note

All claims expressed in this article are solely those of the authors and do not necessarily represent those of their affiliated organizations, or those of the publisher, the editors and the reviewers. Any product that may be evaluated in this article, or claim that may be made by its manufacturer, is not guaranteed or endorsed by the publisher.
